# Nitroglycerine Induced Acute Myocardial Infarction in a Patient with Myocardial Bridging

**DOI:** 10.1155/2014/289879

**Published:** 2014-03-04

**Authors:** Dragana Rujic, Mette Lundgren Nielsen, Karsten Tange Veien, Manan Pareek

**Affiliations:** ^1^Department of Internal Medicine, Odense University Hospital, Svendborg Hospital, Valdemarsgade 53, 5700 Svendborg, Denmark; ^2^Department of Cardiology, Vejle Hospital, Kabbeltoft 25, 7100 Vejle, Denmark; ^3^Department of Cardiology, Odense University Hospital, Sdr. Boulevard 29, 5000 Odense C, Denmark; ^4^Department of Cardiology, Aarhus University Hospital, Skejby, Brendstrupgårdsvej 100, 8200 Aarhus N, Denmark

## Abstract

Muscle overlying an intramyocardial segment of a coronary artery is termed a myocardial bridge. The intramyocardial segment, the tunneled artery, is compressed during systole. The condition is generally benign but may occasionally cause myocardial ischemia, infarction, arrhythmia, or sudden cardiac death. We present a case regarding a 52-year-old man with exercise-induced angina who was diagnosed with a myocardial bridge overlying the left anterior descending artery. He was initially treated with beta-blockers and later received coronary bypass graft surgery.

## 1. Introduction

The major coronary arteries are normally distributed epicardially, that is, on the surface of the myocardium. Occasionally, these vessels have a segmental intramyocardial course. During systole, this segment is compressed either partially or completely. Muscle overlying the intramyocardial segment is called a myocardial bridge, and the artery coursing within the myocardium is termed a tunneled artery [1–3].

## 2. Case Presentation

A 52-year-old man with a long-standing history of smoking and a positive family history of coronary artery disease (CAD) had undergone multiple admissions and investigations in several different hospitals during the last 13 years due to exercise-induced chest pain, shortness of breath, and palpitations.

The electrocardiogram (ECG) showed sinus rhythm with complete right bundle branch block. The concentration of the myocardial tissue-specific biomarker, troponin T, was within the reference range during each hospitalization. A 7-day Holter-monitor recording and an exercise stress test also showed normal results.

A transthoracic echocardiogram showed a slightly increased tricuspid regurgitation jet peak gradient (38 mmHg), and a subsequent computed tomography (CT) of the heart revealed dilatation of the right-sided chambers with a sinus venosus-type atrial septal defect (ASD) with partial anomalous pulmonary venous return (abnormal return of the right upper pulmonary vein into sinus venosus) and poor contrast enhancement of the left anterior descending artery (LAD), but no coronary artery calcification. The anomalous anatomical findings were confirmed by transesophageal echocardiography.

**Figure 1:**
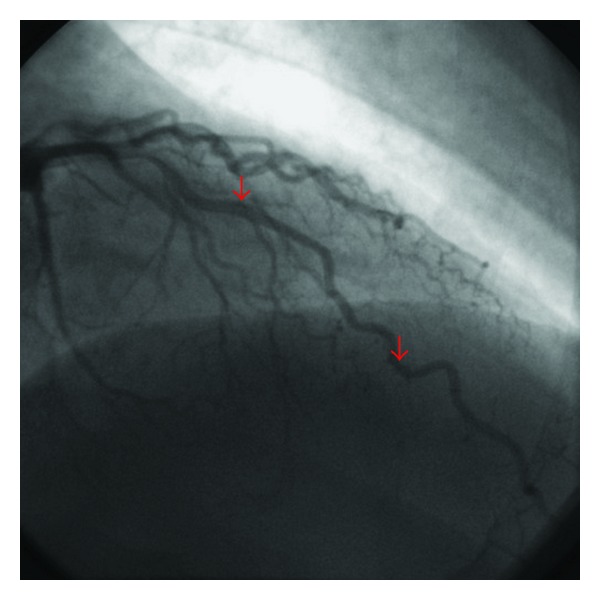
Coronary angiogram showing slight systolic compression of LAD.

**Figure 2:**
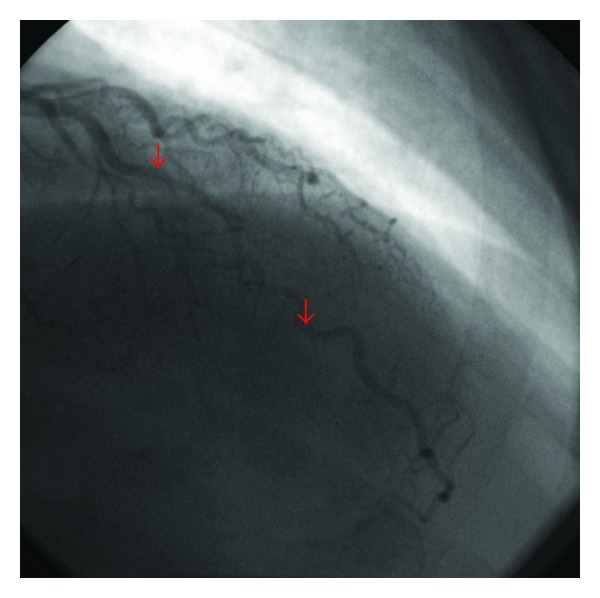
Systolic compression of LAD augmented during nitroglycerin infusion.

To further examine the coronary anatomy, the patient underwent invasive coronary angiography, revealing a myocardial bridge confined to the LAD with mild systolic compression (Figure 1), which worsened during intravenous administration of nitroglycerin (Figure 2). During the angiography, he developed mild chest pain, which continued thereafter in the ward. Despite the angiographic findings, he was given sublingual nitroglycerin, which caused worsening of the symptoms, development of anterior ST-segment elevation in the ECG, and an increased level of high-sensitivity TnT at 686 ng/L (99th percentile 14 ng/L), thus fulfilling the criteria for ST-segment elevation acute myocardial infarction.

Since the coronary anatomy was known, a reangiography was not deemed necessary. The patient was initially treated with metoprolol and aspirin and later underwent surgical closure of the ASD, redirection of the right upper pulmonary vein into the left atrium, and coronary artery bypass surgery (CABG) with the left internal mammary artery (LIMA) to LAD.

## 3. Discussion

Myocardial bridging represents the most common congenital coronary anomaly and most often involves the LAD [1–3]. The prevalence depends greatly on the method of evaluation and is less than 5% in patients undergoing coronary angiography, during which the condition is distinguished from fixed stenoses by showing narrowing of the involved coronary vessels only during systole [1]. In contrast, CT and autopsy studies have reported frequencies of up to 80% [1–3].

The finding of myocardial bridging is usually of little or no clinical significance. The severity, however, varies, and myocardial bridging may lead to myocardial ischemia, myocardial infarction, arrhythmia, or sudden cardiac death [1–3]. Because of the hemodynamic alterations, atherosclerotic changes are often located proximal to the bridge, whereas the tunneled segment itself typically is not atherosclerotic [4–7]. The anatomical changes cannot fully explain the symptoms; however, myocardial bridging augments the risk of significant ischemia during increased sympathetic drive and tachycardia due to a greater systolic-diastolic time ratio and increased contractility [1, 8]. Studies also suggest that a severe systolic compression can, by itself, affect the diastolic blood flow and that the intramyocardial segment may be the source of endothelial dysfunction leading to an increased risk of arterial spasm and thrombosis [1, 5–7].

The diagnosis of myocardial bridging should be considered in younger patients without significant risk factors for CAD, who have exercise-induced angina and possibly perfusion defects detected by appropriate imaging modalities [2]. The systolic compression can be accentuated by careful injection of nitroglycerin during coronary angiography [9, 10].

Medication is considered first-line therapy. Beta-blockers and possibly nondihydropyridine calcium channel blockers are the preferred antianginal agents due to their negative inotropic and chronotropic effects [1–3, 11]. Nitrates may relieve symptoms but are considered contraindicated by most investigators since they reduce the intrinsic coronary wall tension and increase the reflex sympathetic stimulation of contractility [2]. In view of the frequently found atherosclerotic changes proximal to the bridge, antiplatelet drugs and statins may be considered as preventive measures. Invasive treatment strategies should be reserved for high-risk patients and patients with evidence of clinically relevant ischemia and persistent symptoms despite medical therapy [1, 3]. Surgical myotomy (resection of the muscle bridge) or coronary artery bypass surgery (CABG) are preferred over percutaneous coronary intervention (PCI) with stenting because data indicate a high risk of in-stent restenosis [1–3, 12, 13].

The present case illustrates the fact that myocardial bridges are not always benign and may be associated with other congenital heart defects. Clearly shown is also the fact that nitroglycerin, besides diagnostic use, is contraindicated in these patients.
